# Sodium 2-iodo­benzene­sulfonate monohydrate

**DOI:** 10.1107/S1600536808039202

**Published:** 2008-11-29

**Authors:** Muhammad Nadeem Arshad, M. Nawaz Tahir, Islam Ullah Khan, Muhammad Shafiq, Waseeq Ahmad Siddiqui

**Affiliations:** a Department of Chemistry, Government College University, Lahore, Pakistan; b Department of Physics, University of Sargodha, Sargodha, Pakistan; c Department of Chemistry, University of Sargodha, Sargodha, Pakistan

## Abstract

In the title compound, Na^+^·C_6_H_4_IO_3_S^−^·H_2_O, the Na atom is hexa­coordinated by O atoms, forming a two-dimensional sheet-like structure in the *bc* plane, with the iodo­benzene rings protruding above and below. Na⋯O contact distances are in the range 2.419 (2)–2.7218 (18) Å and O⋯Na⋯O angles are in the range 73.70 (5)–158.64 (7)°. The crystal structure is stabilized by O—H⋯O and C—H⋯O hydrogen bonds and C—H⋯π inter­actions. The I atom is disordered over two positions with occupancies of 0.78 (2) and 0.22 (2).

## Related literature

For related literature on the synthesis of biologically active benzothia­zine derivatives, see: Arshad *et al.* (2008 ▶); Chau & Kice (1977 ▶); Shafiq, Khan *et al.* (2008 ▶); Shafiq, Tahir *et al.* (2008 ▶); Tahir *et al.* (2008 ▶).
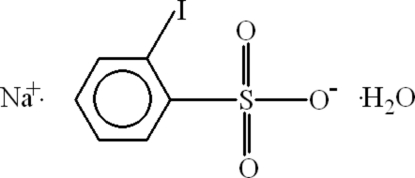


## Experimental

### 

#### Crystal data

Na^+^·C_6_H_4_IO_3_S^−^·H_2_O
*M* *_r_* = 324.06
Monoclinic, 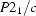

*a* = 13.6141 (4) Å
*b* = 8.8233 (3) Å
*c* = 7.8493 (3) Å
β = 92.171 (1)°
*V* = 942.19 (6) Å^3^
*Z* = 4
Mo *K*α radiation
μ = 3.64 mm^−1^
*T* = 296 (2) K
0.25 × 0.17 × 0.15 mm

#### Data collection

Bruker KAPPA APEXII CCD diffractometer
Absorption correction: multi-scan (*SADABS*; Bruker, 2005 ▶) *T*_min_ = 0.482, *T*_max_ = 0.580
10141 measured reflections
2339 independent reflections
2135 reflections with *I* > 2σ(*I*)
*R*_int_ = 0.024

#### Refinement

*R*[*F* ^2^ > 2σ(*F* ^2^)] = 0.019
*wR*(*F* ^2^) = 0.047
*S* = 1.05
2339 reflections
128 parameters
H-atom parameters constrained
Δρ_max_ = 0.41 e Å^−3^
Δρ_min_ = −0.42 e Å^−3^

### 

Data collection: *APEX2* (Bruker, 2007 ▶); cell refinement: *SAINT* (Bruker, 2007 ▶); data reduction: *SAINT*; program(s) used to solve structure: *SHELXS97* (Sheldrick, 2008 ▶); program(s) used to refine structure: *SHELXL97* (Sheldrick, 2008 ▶); molecular graphics: *ORTEP-3 for Windows* (Farrugia, 1997 ▶) and *PLATON* (Spek, 2003 ▶); software used to prepare material for publication: *WinGX* (Farrugia, 1999 ▶) and *PLATON*.

## Supplementary Material

Crystal structure: contains datablocks global, I. DOI: 10.1107/S1600536808039202/su2075sup1.cif

Structure factors: contains datablocks I. DOI: 10.1107/S1600536808039202/su2075Isup2.hkl

Additional supplementary materials:  crystallographic information; 3D view; checkCIF report

## Figures and Tables

**Table 1:** Hydrogen-bond geometry (Å, °)

*D*—H⋯*A*	*D*—H	H⋯*A*	*D*⋯*A*	*D*—H⋯*A*
O4—H4*A*⋯O2^i^	0.85	1.98	2.824 (2)	174
C6—H6⋯O1	0.93	2.42	2.834 (3)	107
C5—H5⋯*Cg*^i^	0.93	2.79	3.661 (3)	156

Symmetry code: (i) 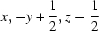. *Cg *is the centroid of the benzene ring.

**Fig. 1.**
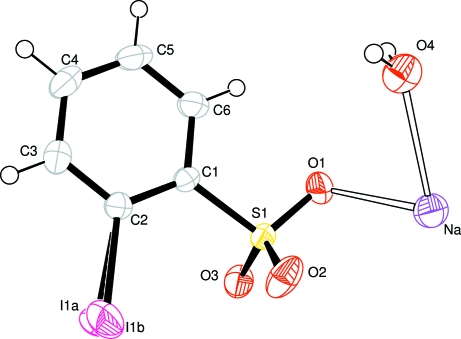
Molecular structure of compound (I), with the atom numbering scheme. The thermal ellipsoids are drawn at the 50% probability level.

**Fig. 2.**
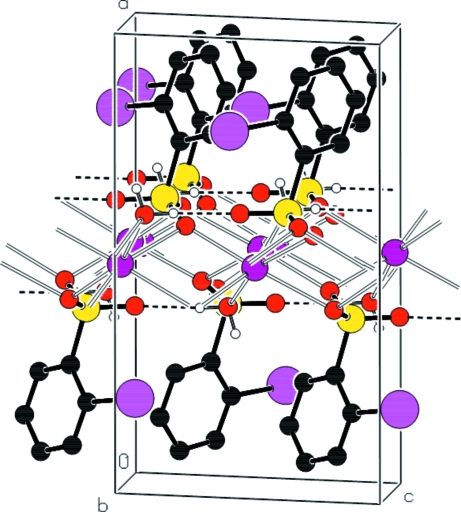
A view along the c axis of the crystal packing in compound (I), showing the coordination of the O-atoms with the sodium atom and the hydrogen bonding involving the water molecules (H-atoms not involved in hydrogen bonding have been removed for clarity).

## supplementary crystallographic information

### Comment

In the continuation to our research work on the synthesis of biologically active benzothiazine derivatives (Arshad *et al.*, 2008; Shafiq, Khan, Tahir & Siddiqui, 2008; Shafiq, Tahir, Khan, Ahmad & Siddiqui, 2008; Tahir *et al.*, 2008), we synthesized the title compound, (I).

The molecular structure of compound (I) is illustrated in Fig. 1. The bond lengths and bond angles, as far as the 2-iodobenzenesulfonate is concerned, are similar to those reported for the potassium salt analogue, reported on recently (Arshad *et al.*, 2008). The Na-atom is hexa-coordinated with O-atoms, involving four from the sulfonic groups and two water molecules. The range of the Na···O distances is 2.419 (2)–2.7218 (18) Å, whereas the range of O···Na···O angles is 73.70 (5)–158.64 (7)°. In this way a two-dimensional sheet-like structure in the bc plane is formed, with the iodobenzene rings protruding above and below.

It is interesting that one of the O-atoms of the sulfonic group is not involved in coordination with the Na-atom, but makes an intermolecular hydrogen bond with a water molecule, similar to the situation on the potassium salt analogue mentioned above. Also, one of the H-atoms of the H_2_O molecule is not involved in intra or intermolecular H-bonding.

In the crystal structure of compound (I) a two dimensional polymeric network extending along the *b* axis is formed (Fig. 2). There exist O-H···O and C-H···O hydrogen bonds, and C-H···π-interactions involving the centroid, *Cg*, of the benzene ring, see Table 1 for details.

### Experimental

The title compound was prepared following the method used by Chau & Kice (1977). Suitable crystals for X-ray analysis were obtained from the reaction mixture.

### Refinement

The Iodine atom was disordered over two positions (I1A/I1B) with occupancies of 0.78 (2)/0.22 (2). The water H-atoms were located in a difference Fourier map and were refined with distance restraints: O-H = 0.82 (2) Å with *U*_iso_(H) = 1.2U_eq_(O). The C-bound H-atoms were positioned geometrically and treated as riding atoms: C—H = 0.93 Å and *U*_iso_(H) = 1.2U_eq_(C).

### Crystal data

**Table:**

Na^+^·C_6_H_4_IO_3_S^–^·H2O	*F*_000_ = 616
*M**_r_* = 324.06	*D*_x_ = 2.285 Mg m^−^^3^
Monoclinic, *P*2_1_/*c*	Mo *K*α radiation λ = 0.71073 Å
Hall symbol: -P 2ybc	Cell parameters from 2339 reflections
*a* = 13.6141 (4) Å	θ = 1.5–28.3º
*b* = 8.8233 (3) Å	µ = 3.64 mm^−^^1^
*c* = 7.8493 (3) Å	*T* = 296 (2) K
β = 92.171 (1)º	Prismatic, colorless
*V* = 942.19 (6) Å^3^	0.25 × 0.17 × 0.15 mm
*Z* = 4	

### Data collection

**Table:**

Bruker KAPPA APEXII CCD diffractometer	2339 independent reflections
Radiation source: fine-focus sealed tube	2135 reflections with *I* > 2σ(*I*)
Monochromator: graphite	*R*_int_ = 0.024
Detector resolution: 7.40 pixels mm^-1^	θ_max_ = 28.3º
*T* = 296(2) K	θ_min_ = 1.5º
ω scans	*h* = −18→18
Absorption correction: multi-scan(SADABS; Bruker, 2005)	*k* = −11→11
*T*_min_ = 0.482, *T*_max_ = 0.580	*l* = −10→10
10141 measured reflections	

### Refinement

**Table:**

Refinement on *F*^2^	Secondary atom site location: difference Fourier map
Least-squares matrix: full	Hydrogen site location: inferred from neighbouring sites
*R*[*F*^2^ > 2σ(*F*^2^)] = 0.019	H-atom parameters constrained
*wR*(*F*^2^) = 0.047	*w* = 1/[σ^2^(*F*_o_^2^) + (0.0176*P*)^2^ + 0.7234*P*] where *P* = (*F*_o_^2^ + 2*F*_c_^2^)/3
*S* = 1.05	(Δ/σ)_max_ = 0.009
2339 reflections	Δρ_max_ = 0.42 e Å^−^^3^
128 parameters	Δρ_min_ = −0.42 e Å^−^^3^
Primary atom site location: structure-invariant direct methods	Extinction correction: none

### Special details

**Table:**

Geometry. Bond distances, angles *etc*. have been calculated using the rounded fractional coordinates. All su's are estimated from the variances of the (full) variance-covariance matrix. The cell e.s.d.'s are taken into account in the estimation of distances, angles and torsion angles
Refinement. Refinement of *F*^2^ against ALL reflections. The weighted *R*-factor *wR* and goodness of fit *S* are based on *F*^2^, conventional *R*-factors *R* are based on *F*, with *F* set to zero for negative *F*^2^. The threshold expression of *F*^2^ > σ(*F*^2^) is used only for calculating *R*-factors(gt) *etc*. and is not relevant to the choice of reflections for refinement. *R*-factors based on *F*^2^ are statistically about twice as large as those based on *F*, and *R*- factors based on ALL data will be even larger.

### Fractional atomic coordinates and isotropic or equivalent isotropic displacement parameters (Å^2^)

**Table:**

	*x*	*y*	*z*	*U*_iso_*/*U*_eq_	Occ. (<1)
I1A	0.19343 (9)	−0.1923 (2)	0.55095 (18)	0.0385 (2)	0.78 (2)
I1B	0.1984 (5)	−0.1716 (17)	0.5644 (12)	0.0628 (13)	0.22 (2)
S1	0.37585 (3)	0.02639 (5)	0.34970 (6)	0.0236 (1)	
Na	0.51091 (7)	0.29771 (10)	0.49492 (12)	0.0402 (3)	
O1	0.42883 (11)	0.14214 (18)	0.2606 (2)	0.0369 (5)	
O2	0.38308 (12)	0.0514 (2)	0.53146 (19)	0.0461 (6)	
O3	0.40017 (11)	−0.12539 (18)	0.2976 (2)	0.0390 (5)	
O4	0.39172 (13)	0.4952 (2)	0.38765 (19)	0.0413 (5)	
C1	0.24956 (13)	0.0530 (2)	0.2877 (2)	0.0227 (5)	
C2	0.17328 (14)	−0.0292 (2)	0.3573 (2)	0.0251 (5)	
C3	0.07722 (15)	−0.0064 (3)	0.2991 (3)	0.0328 (6)	
C4	0.05587 (16)	0.0976 (3)	0.1724 (3)	0.0386 (7)	
C5	0.13033 (18)	0.1803 (3)	0.1044 (3)	0.0412 (7)	
C6	0.22673 (16)	0.1593 (3)	0.1618 (3)	0.0332 (6)	
H3	0.02673	−0.06165	0.34590	0.0393*	
H4	−0.00875	0.11181	0.13299	0.0463*	
H4A	0.38645	0.48643	0.28018	0.0496*	
H4B	0.33495	0.51683	0.41658	0.0496*	
H5	0.11586	0.25088	0.01914	0.0495*	
H6	0.27653	0.21667	0.11578	0.0398*	

### Atomic displacement parameters (Å^2^)

**Table:**

	*U*^11^	*U*^22^	*U*^33^	*U*^12^	*U*^13^	*U*^23^
I1A	0.0425 (3)	0.0384 (5)	0.0345 (3)	−0.0046 (4)	0.0010 (2)	0.0146 (3)
I1B	0.0625 (16)	0.071 (3)	0.0543 (18)	−0.0264 (19)	−0.0055 (11)	0.0310 (15)
S1	0.0214 (2)	0.0248 (2)	0.0243 (2)	0.0002 (2)	−0.0019 (2)	−0.0017 (2)
Na	0.0466 (5)	0.0325 (5)	0.0407 (5)	0.0011 (4)	−0.0087 (4)	−0.0053 (4)
O1	0.0285 (7)	0.0346 (8)	0.0478 (9)	−0.0059 (6)	0.0027 (6)	0.0055 (7)
O2	0.0349 (8)	0.0784 (13)	0.0245 (7)	−0.0004 (8)	−0.0055 (6)	−0.0061 (8)
O3	0.0301 (8)	0.0252 (8)	0.0612 (10)	0.0051 (6)	−0.0043 (7)	−0.0076 (7)
O4	0.0447 (9)	0.0519 (10)	0.0274 (7)	0.0000 (8)	0.0018 (7)	−0.0009 (7)
C1	0.0224 (8)	0.0227 (9)	0.0227 (8)	0.0025 (7)	−0.0012 (7)	−0.0016 (7)
C2	0.0277 (9)	0.0240 (9)	0.0234 (9)	0.0001 (7)	−0.0006 (7)	−0.0011 (7)
C3	0.0261 (9)	0.0349 (12)	0.0373 (11)	−0.0030 (8)	0.0008 (8)	−0.0037 (9)
C4	0.0283 (10)	0.0430 (13)	0.0438 (12)	0.0083 (9)	−0.0091 (9)	−0.0036 (10)
C5	0.0405 (12)	0.0413 (14)	0.0413 (12)	0.0096 (10)	−0.0066 (10)	0.0141 (10)
C6	0.0333 (10)	0.0316 (11)	0.0346 (11)	0.0019 (8)	0.0017 (8)	0.0091 (9)

### Geometric parameters (Å, °)

**Table:**

I1A—C2	2.104 (2)	O4—H4B	0.8400
I1B—C2	2.073 (12)	O4—H4A	0.8500
S1—O1	1.4459 (16)	C1—C6	1.389 (3)
S1—O2	1.4433 (16)	C1—C2	1.395 (3)
S1—O3	1.4424 (16)	C2—C3	1.384 (3)
S1—C1	1.7846 (18)	C3—C4	1.376 (4)
Na—O1	2.5219 (18)	C4—C5	1.373 (3)
Na—O4	2.505 (2)	C5—C6	1.384 (3)
Na—O3^i^	2.7218 (18)	C3—H3	0.9300
Na—O3^ii^	2.5060 (18)	C4—H4	0.9300
Na—O4^iii^	2.419 (2)	C5—H5	0.9300
Na—O1^iv^	2.4609 (18)	C6—H6	0.9300
			
I1A···O2	3.368 (2)	O4···O1^iv^	3.191 (2)
I1A···O3	3.557 (2)	O4···O1^i^	3.036 (2)
I1A···C1^v^	3.750 (2)	O1···H4A^viii^	2.8900
I1A···I1A^vi^	4.055 (2)	O1···H6	2.4200
I1A···I1A^v^	4.055 (2)	O2···H6^iv^	2.6100
I1A···C2^v^	3.456 (2)	O2···H4A^iv^	1.9800
I1A···C3^v^	3.688 (3)	C1···I1A^vi^	3.750 (2)
I1B···O3	3.540 (8)	C1···I1B^vi^	3.846 (14)
I1B···O2	3.211 (11)	C2···I1A^vi^	3.456 (2)
I1B···C3^v^	3.797 (13)	C2···I1B^vi^	3.526 (13)
I1B···C1^v^	3.846 (14)	C3···I1A^vi^	3.688 (3)
I1B···C2^v^	3.526 (13)	C3···I1B^vi^	3.797 (13)
I1A···H4^vii^	3.3300	C4···C4^x^	3.506 (3)
S1···O2^ii^	3.4468 (17)	C2···H5^iv^	2.8900
O1···O3^i^	3.149 (2)	C3···H5^iv^	2.8800
O1···O4^viii^	3.036 (2)	C4···H4^x^	3.0700
O1···O4^ix^	3.191 (2)	C6···H4B^ix^	2.9200
O2···S1^ii^	3.4468 (17)	H4···I1A^xi^	3.3300
O2···I1B	3.211 (11)	H4···C4^x^	3.0700
O2···I1A	3.368 (2)	H4A···O2^ix^	1.9800
O2···O4^iv^	2.824 (2)	H4B···C6^iv^	2.9200
O3···I1B	3.540 (8)	H5···C2^ix^	2.8900
O3···O1^viii^	3.149 (2)	H5···C3^ix^	2.8800
O3···I1A	3.557 (2)	H6···O1	2.4200
O4···O2^ix^	2.824 (2)	H6···O2^ix^	2.6100
			
O1—S1—O2	110.70 (10)	Na—O4—Na^iii^	93.37 (7)
O1—S1—O3	113.24 (9)	Na^iii^—O4—H4A	118.00
O1—S1—C1	105.56 (9)	Na^iii^—O4—H4B	103.00
O2—S1—O3	114.48 (10)	Na—O4—H4A	107.00
O2—S1—C1	106.16 (9)	Na—O4—H4B	131.00
O3—S1—C1	105.90 (9)	H4A—O4—H4B	104.00
O1—Na—O4	82.53 (6)	C2—C1—C6	118.68 (17)
O1—Na—O3^i^	73.70 (5)	S1—C1—C6	118.04 (14)
O1—Na—O3^ii^	109.48 (6)	S1—C1—C2	123.27 (13)
O1—Na—O4^iii^	155.53 (7)	I1A—C2—C3	115.71 (15)
O1—Na—O1^iv^	122.18 (6)	I1A—C2—C1	124.06 (14)
O3^i^—Na—O4	81.15 (6)	I1B—C2—C3	118.2 (2)
O3^ii^—Na—O4	158.64 (7)	C1—C2—C3	120.23 (17)
O4—Na—O4^iii^	86.63 (6)	I1B—C2—C1	121.3 (2)
O1^iv^—Na—O4	79.96 (6)	C2—C3—C4	120.5 (2)
O3^i^—Na—O3^ii^	118.69 (6)	C3—C4—C5	119.8 (2)
O3^i^—Na—O4^iii^	83.01 (6)	C4—C5—C6	120.5 (2)
O1^iv^—Na—O3^i^	153.11 (6)	C1—C6—C5	120.4 (2)
O3^ii^—Na—O4^iii^	88.08 (6)	C2—C3—H3	120.00
O1^iv^—Na—O3^ii^	78.69 (6)	C4—C3—H3	120.00
O1^iv^—Na—O4^iii^	76.94 (6)	C3—C4—H4	120.00
S1—O1—Na	104.30 (8)	C5—C4—H4	120.00
S1—O1—Na^ix^	144.40 (10)	C4—C5—H5	120.00
Na—O1—Na^ix^	107.32 (6)	C6—C5—H5	120.00
S1—O3—Na^viii^	125.79 (9)	C1—C6—H6	120.00
S1—O3—Na^ii^	119.31 (9)	C5—C6—H6	120.00
Na^viii^—O3—Na^ii^	100.23 (6)		
			
O2—S1—O1—Na	4.83 (11)	O3^i^—Na—O4—Na^iii^	−83.44 (6)
O2—S1—O1—Na^ix^	156.95 (15)	O3^ii^—Na—O4—Na^iii^	75.95 (19)
O3—S1—O1—Na	−125.30 (9)	O4^iii^—Na—O4—Na^iii^	0.00 (7)
O3—S1—O1—Na^ix^	26.83 (19)	O1^iv^—Na—O4—Na^iii^	77.33 (6)
C1—S1—O1—Na	119.29 (8)	O1—Na—O3^i^—S1^i^	136.29 (11)
C1—S1—O1—Na^ix^	−88.58 (16)	O1—Na—O3^i^—Na^ix^	−1.72 (6)
O1—S1—O3—Na^viii^	−18.05 (13)	O4—Na—O3^i^—S1^i^	51.55 (11)
O1—S1—O3—Na^ii^	112.93 (10)	O4—Na—O3^i^—Na^ix^	−86.47 (6)
O2—S1—O3—Na^viii^	−146.24 (10)	O1—Na—O3^ii^—S1^ii^	19.44 (12)
O2—S1—O3—Na^ii^	−15.26 (13)	O1—Na—O3^ii^—Na^iv^	−122.08 (6)
C1—S1—O3—Na^viii^	97.16 (10)	O4—Na—O3^ii^—S1^ii^	141.17 (16)
C1—S1—O3—Na^ii^	−131.86 (9)	O4—Na—O3^ii^—Na^iv^	−0.4 (2)
O1—S1—C1—C2	−174.65 (15)	O1—Na—O4^iii^—Na^iii^	63.66 (18)
O1—S1—C1—C6	6.44 (18)	O4—Na—O4^iii^—Na^iii^	0.00 (6)
O2—S1—C1—C2	−57.08 (17)	O1—Na—O1^iv^—S1^iv^	−100.39 (17)
O2—S1—C1—C6	124.01 (17)	O1—Na—O1^iv^—Na^iv^	107.94 (8)
O3—S1—C1—C2	65.01 (16)	O4—Na—O1^iv^—S1^iv^	−25.90 (16)
O3—S1—C1—C6	−113.91 (17)	O4—Na—O1^iv^—Na^iv^	−177.57 (7)
O4—Na—O1—S1	−111.84 (9)	S1—C1—C2—I1A	2.0 (2)
O4—Na—O1—Na^ix^	84.73 (7)	S1—C1—C2—C3	−177.78 (16)
O3^i^—Na—O1—S1	165.24 (9)	C6—C1—C2—I1A	−179.11 (16)
O3^i^—Na—O1—Na^ix^	1.81 (6)	C6—C1—C2—C3	1.1 (3)
O3^ii^—Na—O1—S1	49.96 (10)	S1—C1—C6—C5	177.54 (18)
O3^ii^—Na—O1—Na^ix^	−113.48 (7)	C2—C1—C6—C5	−1.4 (3)
O4^iii^—Na—O1—S1	−176.30 (14)	I1A—C2—C3—C4	−179.9 (2)
O4^iii^—Na—O1—Na^ix^	20.27 (19)	C1—C2—C3—C4	−0.1 (3)
O1^iv^—Na—O1—S1	−38.71 (11)	C2—C3—C4—C5	−0.6 (4)
O1^iv^—Na—O1—Na^ix^	157.85 (7)	C3—C4—C5—C6	0.3 (4)
O1—Na—O4—Na^iii^	−158.02 (6)	C4—C5—C6—C1	0.7 (4)

Symmetry codes: (i) −*x*+1, *y*+1/2, −*z*+1/2; (ii) −*x*+1, −*y*, −*z*+1; (iii) −*x*+1, −*y*+1, −*z*+1; (iv) *x*, −*y*+1/2, *z*+1/2; (v) *x*, −*y*−1/2, *z*+1/2; (vi) *x*, −*y*−1/2, *z*−1/2; (vii) −*x*, *y*−1/2, −*z*+1/2; (viii) −*x*+1, *y*−1/2, −*z*+1/2; (ix) *x*, −*y*+1/2, *z*−1/2; (x) −*x*, −*y*, −*z*; (xi) −*x*, *y*+1/2, −*z*+1/2.

### Hydrogen-bond geometry (Å, °)

**Table:**

*D*—H···*A*	*D*—H	H···*A*	*D*···*A*	*D*—H···*A*
O4—H4A···O2^ix^	0.85	1.98	2.824 (2)	174
C6—H6···O1	0.93	2.42	2.834 (3)	107
C5—H5···Cg^ix^	0.93	2.79	3.661 (3)	156

Symmetry codes: (ix) *x*, −*y*+1/2, *z*−1/2.
